# Effectiveness of 12‐week inspiratory muscle training with manual therapy in patients with COPD: A randomized controlled study

**DOI:** 10.1111/crj.13486

**Published:** 2022-03-24

**Authors:** Yasemin Buran Cirak, Gul Deniz Yilmaz Yelvar, Nurgül Durustkan Elbasi

**Affiliations:** ^1^ Physiotherapy and Rehabilitation Department, Faculty of Health Science Istinye University Istanbul Turkey

**Keywords:** COPD, functional capacity, inspiratory muscle training, manual therapy

## Abstract

The benefits of inspiratory muscle training (IMT) in patients with COPD were reported. However, its effects are limited in severe COPD patients. Further researches are required in new and complementary modalities demonstrating IMT efficacy in severe COPD patients. This study aims to investigate effects of manual therapy (MT) additional over IMT on functional capacity, respiratory muscle strength, pulmonary function, dyspnea, fatigue, and quality of life in severe COPD patients. Sixty patients with COPD in GOLD stage III–IV were included in this prospective single‐blind randomized trial. Patients were randomly assigned to receive either MT additional over IMT at 40% of maximal inspiratory pressure (MIP) (*n* = 30) or only IMT (*n* = 30) for 12 weeks. MT group received MT during 12 weeks for 30 min additional to IMT. Pulmonary function, respiratory muscle strength, functional capacity, dyspnea, fatigue, and quality of life were evaluated by spirometry, mouth pressure device, six‐minute walk test, Modified Medical Research Council (mMRC) dyspnea scale, fatigue severity scale, and St. George's Respiratory Questionnaire (SGRQ), respectively. MT group had significantly greater improvement in FEV1%, FVC%, PEF%, respiratory muscle strength, function, dyspnea, fatigue, and quality of life compared with IMT group (*p* < 0.05). 6MWT (*p* < 0.001, effect size Cohen's *d*: 0.915), MIP (*p* < 0.001, effect size Cohen's *d*: 1.235), and mMRC score (*p* < 0.001, effect size Cohen's *d*: 0.982) were significantly improved in IMT with MT group. This study demonstrated that subjects in IMT with MT group had improved outcomes in functional capacity, respiratory muscle strength, pulmonary function, dyspnea, fatigue perception, and quality of life compared with alone IMT group.

## Graphical abstract

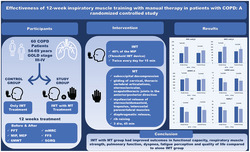

## 1 INTRODUCTİON

Chronic obstructive pulmonary disease (COPD) is a rising universal problem. Extrapulmonary comorbidities are common in COPD and significantly contribute to mortality, symptoms, exacerbations, and hospital admissions. The frequency of exacerbation and the presence and severity of symptoms have been stated as two important factors in the progression of COPD disease. Therefore, reduction of symptoms and exacerbation will slow down the progression of the disease. Also, the restructured classical pulmonary system‐centered definition of the Global Initiative for Chronic Obstructive Lung Disease (GOLD) emphasizes the importance of extrapulmonary conditions in COPD patients and their clinics.^1^ Although cardiac, metabolic, musculoskeletal, and psychological conditions are among these extrapulmonary conditions, musculoskeletal dysfunction (32%) is one of the most common extrapulmonary comorbidities in patients with COPD.^2^ Respiratory dysfunction in patients with COPD has been associated with compensatory alterations in the length and mobility of the chest, thoracic spine, and shoulder muscles.^3^ Dysfunctions such as inspiratory muscle weakness, reduced respiratory muscle endurance, changes in chest wall mechanics and the position of the diaphragm due to hyperinflation, increased tension of the respiratory muscles, and spinal hypomobility cause impaired exercise capacity, the increased dyspnea, increased effort required to breathe, and poorer quality of life.^4^, ^5^, ^6^

Reducing dyspnea is one of the most important and prioritized targets of the treatment because the onset of dyspnea is the primary reason for exercise cessation. To this end, management strategies such as exercise and inspiratory muscle training (IMT) are used; IMT has been applied for over 30 years to reduce dyspnea and improve inspiratory muscle strength and endurance and overall quality of life.^7^, ^8^ Evidence also suggests that manual therapy (MT) has the potential to alter respiratory mechanics in certain chronic respiratory diseases and there are published studies describing the use of MT techniques, especially osteopathic techniques. MT covers a range of techniques, including soft tissue therapy and joint mobilization/manipulation. Increasing thoracic mobility with MT techniques may work to reduce the work of breathing through enhanced oxygen transport and lymphatic return, while enhanced local circulation, improved nutrition of tissues, and muscle relaxation help generate much more respiratory muscle force.^9^, ^10^ Also, the immediate positive immediate effect of MT on dyspnea and oxygen saturation has been shown in COPD patients after a single MT session.^11^ However, Heneghan et al., the findings of this review, contradict the earlier conclusion that there was no evidence to support or refute the benefits of MT on patients with COPD.^12^ Simonelli et al. investigated the effect of MT alone or combined with exercise training on pulmonary function, functional capacity, and quality of life in COPD patients, and their systematic review found no evidence to support MT.^13^ On the other hand, Wearing et al. reported that MT combined with exercise training improved pulmonary function and exercise capacity.^14^

MT applications to improve respiratory mechanics may help to improve the length‐tension relationship of respiratory muscles, thus supporting strength development by contributing to inspiratory muscle training. To our knowledge, there have been no reports that investigated the effects of IMT combined with MT on patients with COPD. The hypothesis underlying this study was that a 12‐week IMT and MT program increases pulmonary function, inspiratory muscle strength and functional capacity and decreases dyspnea and fatigue perception in patients with COPD.

## 2 METHODS

### 2.1 Design

**FIGURE 1:**
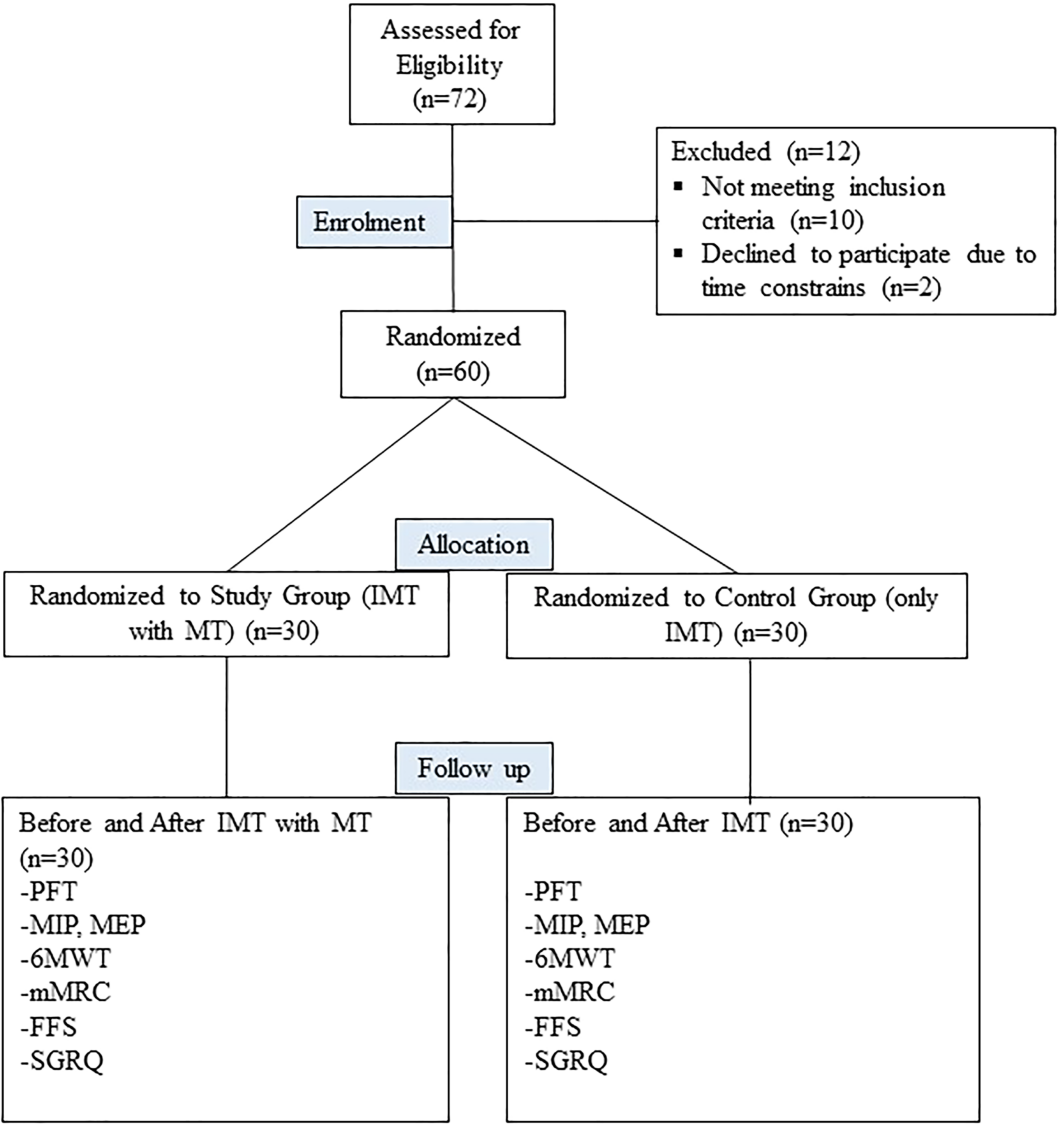
Flowchart of the study

A prospective, randomized controlled, single‐blinded study was performed (clinicaltrials.gov number: NCT04533516). Ethical approval was given by Fatih University Clinical Research Ethics Committee. The study was conducted according to the Declaration of Helsinki and its guidelines for Good Clinical Practice. Participants signed an informed consent form to participate in the study and were randomly divided into two groups. The randomization was made using a randomization table created by a web‐based computer program. The participants were assigned to a study group and a control group in a 1:1 ratio (Figure 1). Group assignments were sealed in sequentially numbered opaque envelopes by a research assistant who was not involved in data collection. Another independent research assistant explained the group assignments to each participant after the first assessment. The investigator collecting pre‐ and post‐treatment data was aware of each participant's group allocation. Patients' evaluations and treatments were performed by a different investigator. Treatments and check‐ups took place at different locations.

### 2.2 Participants

The study was conducted in the Department of Respiratory Medicine and the Department of Cardiopulmonary Physiotherapy at the university hospital. Seventy‐two patients with COPD were recruited from the university hospital. The criterion for patients to be accepted to the study included a clinically stable condition and a forced expiratory volume in 1 s (FEV1) of <50% of the predicted value after bronchodilator drugs. Potential subjects were considered if they had a known history of COPD and an office pulmonary function screening. Diagnosis of disease and classification of disease severity were established in line with the Global Initiative for Chronic Obstructive Lung Disease criteria (GOLD). The study's exclusion criteria included an unstable medical condition, acute bronchitis, pneumonia, exacerbation of COPD, thoracic spinal scoliosis, substantial chest wall deformity, or acute rib or vertebral fracture. Furthermore, subjects were excluded if they were unable to perform the pulmonary function test because of cognitive or physical impairments.

#### 2.2.1 Sample size

Before the participants were included in the study, G*Power 3.0.10. program was used to determine the sample size. In determining the sample size, the MIP value was taken into account among our outcome measurements. In the literature review, the minimum amount of clinically significant change in MIP value as a result of IMT in COPD patients was given as +13 cm H₂O.^7^ Accordingly this reference, to detect the sample size of our study +13 cm H_2_O change with 95% confidence level and 95% power, the study and control groups were determined as 30 patients, in total 60 patients.

Of 72 COPD patients referred by physicians for physiotherapy and 60 patients were selected after applying the inclusion and exclusion criteria. Participants were randomly divided into either a study group (30 patients) or a control group (30 patients). All patients were taking optimal medical treatment. For 12 weeks, the study group received IMT with MT, and the control group received IMT alone. The flow chart for enrollment and testing is shown in Figure 1.

### 2.3 Outcome measures

The main outcome was the measurement of pulmonary function, inspiratory muscle strength, functional capacity, and dyspnea. Secondary outcomes were fatigue perception and quality of life. All measurements were made before and after 12 weeks of treatment.

#### 2.3.1 Pulmonary function tests

Before the pulmonary function test, all subjects rested to avoid fatigue. Pulmonary function measurement was performed using portable spirometry (Spirobank MIR, Italy) according to ATS/ERS criteria in a seated position.^15^, ^16^ FEV_1_, forced vital capacity (FVC), FEV_1_/FVC ratio and peak expiratory flow (PEF), and forced expiratory flow at 25−75% (FEF_25−75_) were expressed as the percentages of the predicted values.

#### 2.3.2 Respiratory muscle strength

In our study, respiratory muscle strength measurement was performed using a portable electronic mouth‐pressure measuring device (Micro Medical MicroMPM, UK). Maximal inspiratory pressure (MIP) is the intraoral pressure measured during maximum inspiration against a valve that closes the airway. In MIP measurement, the respiratory tract is closed with a valve after maximum expiration, and the person is asked to make maximum inspiration and maintain it. Maximal expiratory pressure (MEP) is the intraoral pressure measured during maximum expiration against a valve closing the airway. In MEP measurement, the respiratory tract is closed with a valve after maximal inspiration, and the person is asked to make maximal expiration against the closed respiratory tract. The best of the three measurements is chosen.^17^ In the interpretation of the measurements, the Black and Hyatt equations were used for reference.^18^

#### 2.3.3 Six‐minute walk test (6MWT)

To determine the functional capacity of the participants, 6MWT was applied twice on the same day, half an hour apart. Oxygen saturation values, heart rate, respiratory frequency values, modified Borg scale scores for fatigue, and dyspnea perception were recorded before and after each test. The subjects were asked to walk as fast as possible for 6 min along a straight corridor of 30 m. Before starting the test, the patients were told if they felt too breathless during the test, they could rest and this period would be included in the test. Everyone minute during the test, participants were told, “You are doing well,” in order to encourage them. Oxygen saturation (SpO_2_) and heart rate were monitored throughout the test with a portable pulse oximeter. At the end of the test, the 6MWT distance was recorded. The longer distance value of the two tests was taken.^19^

#### 2.3.4 Modified Medical Research Council (mMRC) Dyspnea Scale

A modified version of the British Medical Research Council (MRC) Dyspnea Scale, the mMRC, is a five‐category (0–4) scale based on various physical activities that cause dyspnea. Patients are asked to mark the activity level where they experience dyspnea.^20^

#### 2.3.5 Fatigue Severity Scale (FSS)

The Fatigue Severity Scale (FSS) is a self‐administered questionnaire consisting of nine items. Each item is scored between 1 and 7, and a lower total score indicates a decrease in fatigue.^21^ This study used the Turkish version of the FSS to evaluate fatigue perception.

#### 2.3.6 Saint George's Respiratory Questionnaire (SGRQ)

Saint George's Respiratory Questionnaire (SGRQ) is a standardized self‐administered questionnaire for pulmonary diseases developed by Jones et al. in 1991.^22^ It has 50 items with 76 weighted responses and three subscales, including symptoms (eight items), activity (16 items), and impacts (26 items). Each subscale and the total questionnaire are scored between “0 and 100,” where “0” indicates the best quality of life and “100” the worst quality of life.^23^ This study used the Turkish version of SGRQ was used to evaluate the quality of life of the participants.

### 2.4 Intervention

#### 2.4.1 IMT protocol

Before IMT, a physiotherapist gave study participants general information about inspiratory muscle training. The physiotherapist administered IMT to all patients using the Threshold IMT device (Respironics, USA). The intensity of IMT was set to 40% of the initial MIP. Patients were asked to train twice every day for 15 min for 12 weeks. They were told to record their practice daily in an official log for the study. The patients came for scheduled monitoring each week and their log records were checked, MIP values measured, and training intensity set at 40% of the new MIP value. IMT was performed under supervision on the monitoring day and was performed at home on the other days. Patients were asked to sit in a relaxed position with their upper chest, shoulders, and arms supported. The nose clip was attached, and the patient was asked to take the mouthpiece of the device into his mouth, close his lips tightly to breathe slowly with an increased tidal volume, and then exhale this breath completely with minimal effort. Patients were instructed to maintain diaphragmatic breathing. After the “10–15” breathing with Threshold IMT device, patients were asked to perform three to five breaths without the device to promote relaxation. Patients were asked to continue this cycle for 15 min at a time. In each supervision session, symptoms such as dizziness, fatigue, and shortness of breath were carefully watched for, and heart rate and SpO_2_ were measured using a pulse oximeter.

#### 2.4.2 MT protocol

In addition to IMT, the same physiotherapist applied MT to the patients in the study group 3 days a week for 12 weeks, during which time patients continued their routine medical treatments. The MT protocol sessions lasted ~45 min and included the following MT techniques:

- suboccipital decompression,
- gliding of the cervical vertebral articulations in the anterior/posterior direction,
- myofascial release of sternocleidomastoid and trapezius muscles,
- gliding of sternoclavicular joint in the anterior/posterior direction,
- myofascial release of intercostal muscles and paravertebral muscles,
- diaphragmatic release,
- rib raising,
- mobilization of scapulothoracic joint, and
- gliding of the thoracic vertebral articulations in the anterior/posterior direction.

The myofascial release techniques were each applied for ~3–5 min. The gliding techniques were performed five times in each joint for 30 s.^10^, ^24^

### 2.5 Statistical analysis

All statistical analyses were performed using the SPSS 22.0 statistical package (SPSS Inc., USA). The Kolmogorov–Smirnov/Shapiro–Wilk test was used to investigate the normality of the distribution of the continuous variables. Continuous variables were normally distributed. The descriptive statistics were given as the mean ± standard deviation for the continuous variables and as the number of patients and percentage (%) for the categorical variables. Differences in nominal variables between the study group and the control group were tested with the chi‐square test. Comparison of the groups was performed using the Student's *T* test. For comparisons within a group, paired sample *T* test was used. The interaction effect between group and time was assessed using repeated measure analysis of covariance (ANCOVA), with the baseline as the covariate. To describe the differences in the related treatments, the effect size between‐groups differences were calculated using the Cohen's *d* test and classified as small (*d* ≥ 0.20 and <0.50), medium (*d* ≥ 0.50 and <0.80), and large (*d* ≥ 0.80). *p* < 0.05 was regarded as statistically significant.

## 3 RESULTS

**FIGURE 2:**
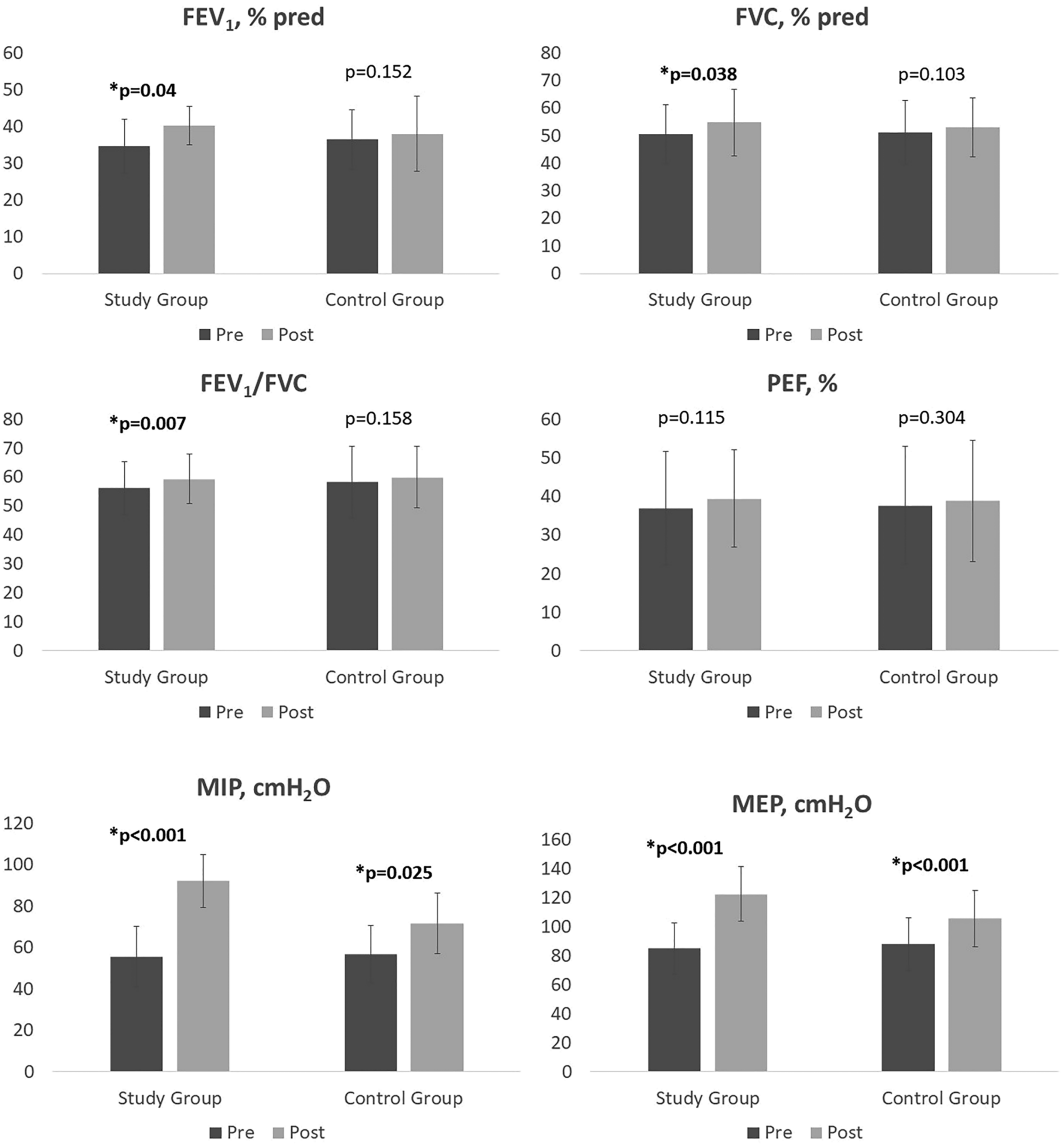
Comparison of treatment effect on pulmonary function and respiratory muscle strength between the groups

**FIGURE 3:**
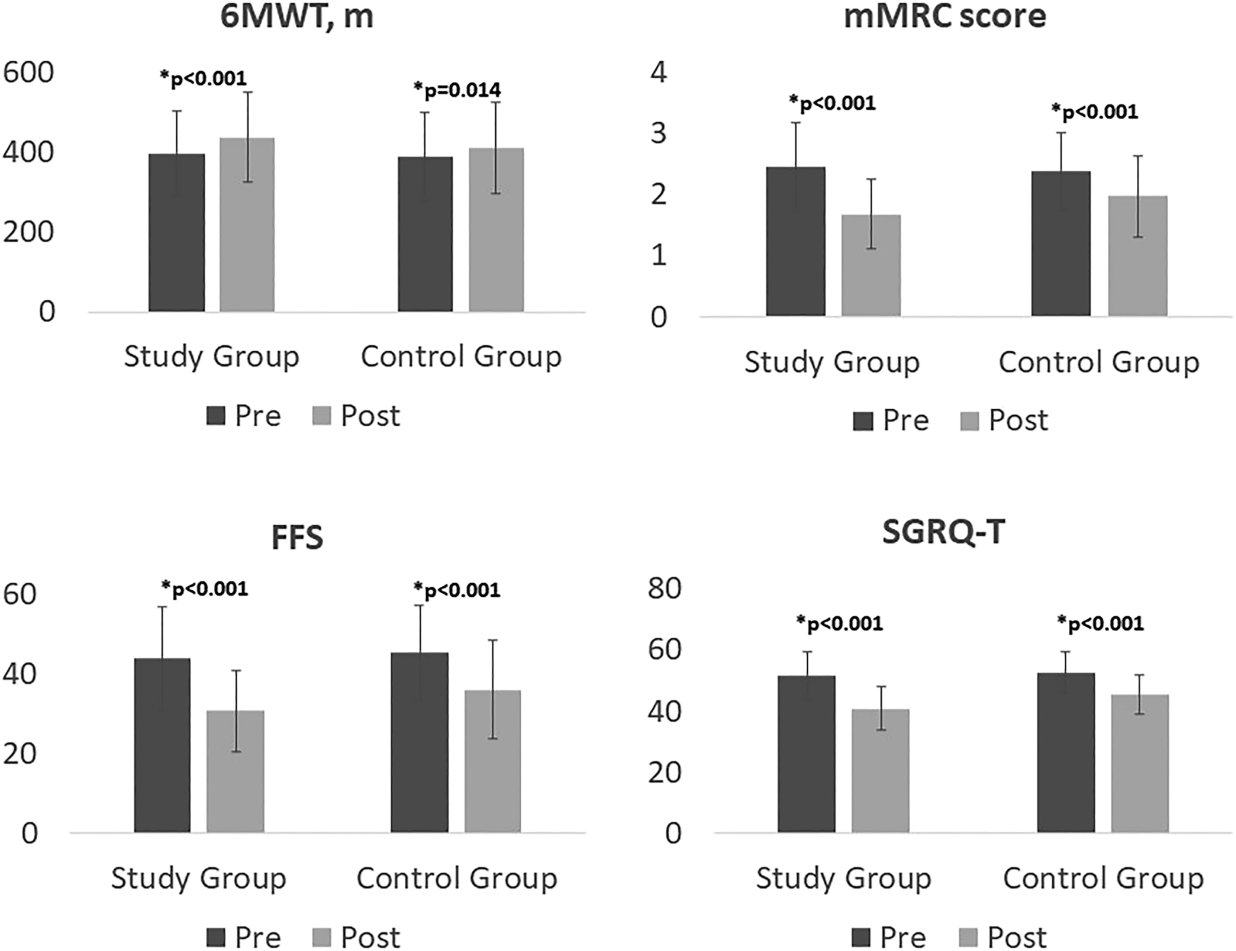
Comparison of treatment effect on functional capacity, dyspnea, fatigue and quality of life between the groups

Sixty patients with COPD at GOLD stage 3 and 4 were included in the study. Thirty patients with COPD were in the study group, and 30 patients with COPD were in the control group. The mean age of the participants in the study group was 62.9 ± 6.5 and 61.2 ± 7.1 in the control group. The disease duration of participants in the study group in years was 6.15 ± 4.82 and 5.36 ± 3.75 in the control group. The study and control group were similar in age, gender, BMI, GOLD stage, smoking exposure, disease duration, number of exacerbations, usage of oxygen therapy, and NIV (*p* > 0.05, Table 1). Before treatment, there was no significant difference in measurements between the study and control groups (*p* > 0.05, Table 2). After treatment, the change in FEV_1_ and FVC values between the study and control groups was significantly different (*p* < 0.05, Table 3), but there was no significant difference in FEV_1_/FVC, PEF, and FEF_25−75_ values between the groups (*p* > 0.05, Table 3). Also after treatment, the change in MIP and MEP values between the study and control groups was significantly different (*p* < 0.05, Table 4, Figure 2). There was significant improvement in functional capacity in both groups but the change in 6MWT distance was greater in the study group (*p* < 0.05, Figure 3). Moreover, after treatment, the change in mMRC and FFS scores between the study and control groups were significantly different (*p* < 0.05, Table 5, Figure 3). When the change in the quality of life between the groups was compared, the change in the total and all subscale scores of SGRQ between the study and control groups were significantly different (*p* < 0.05, Table 6, Figure 3).

**TABLE 1:** The demographic and clinical features of the participants

Variable		Study group (*n* = 30) X ± SS	Control group (*n* = 30) X ± SS	*p*
Age, years		62.9 ± 6.5	61.2 ± 7.1	0.854
BMI, (kg/m^2^)		25.9 ± 4.76	26.4 ± 3.59	0.378
Smoking exposure (pack*year)		47.6 ± 14.5	51.1 ± 16.5	0.119
Disease duration (years)		6.15 ± 4.82	5.36 ± 3.75	0.695
Number of exacerbations		0.75 ± 0.53	0.72 ± 0.86	0.878

		*n* (%)	*n* (%)	
Gender	Female	6 (20)	5 (16.7)	0.762
	Male	24 (80)	25 (83.3)	
Oxygen therapy	Yes	8 (26.7)	7 (23.3)	0.921
	No	22 (73.3)	23 (76.7)	
NIV	Yes	3 (10)	2 (6.7)	0.980
	No	27 (90)	28 (93.7)	
GOLD stage	GOLD stage 3	24 (80)	25 (83.3)	0.842
	GOLD stage 4	6 (20)	5 (16.7)	

*Note*: Statistically significant values are given in bold.

Abbreviations: BMI, body mass index; GOLD, Global Initiative for Chronic Obstructive Lung Disease; NIV, non‐invasive ventilation.

**TABLE 2:** Comparison of all measurements taken before treatment between groups

Variables	Study group (*n* = 30)	Control group (*n* = 30)	*p*
	Mean ± SD	Mean ± SD	
FEV_1_, % pred	34.6 ± 7.33	36.4 ± 8.12	0.769
FVC, % pred	50.4 ± 10.6	51.1 ± 11.6	0.647
FEV_1_/FVC	56.2 ± 9.15	58.2 ± 12.4	0.428
PEF, %	36.8 ± 14.7	37.6 ± 15.2	0.784
FEF_25−75_, %	19.8 ± 10.1	20.1 ± 11.4	0.715
MIP, cmH_2_O	55.5 ± 14.6	56.8 ± 13.8	0.642
MIP, % pred	60.8 ± 18.7	61.2 ± 16.7	0.744
MEP, cmH_2_O	85.1 ± 17.5	88.1 ± 18.3	0.586
MEP, % pred	50.8 ± 20.1	52.1 ± 21.5	0.632
6MWT, m	395.1 ± 106.8	386.7 ± 110.4	0.135
6MWT, % pred	59.4 ± 14.7	58.7 ± 13.8	0.168
mMRC score	2.45 ± 0.72	2.38 ± 0.62	0.334
FFS	43.7 ± 12.7	45.1 ± 11.8	0.251
SGRQ‐S	62.1 ± 12.4	62.3 ± 12.4	0.943
SGRQ‐A	60.3 ± 14.8	61.5 ± 15.1	0.863
SGRQ‐I	40.5 ± 5.68	40.7 ± 7.23	0.947
SGRQ‐T	51.6 ± 7.74	52.6 ± 6.77	0.764

*Note*: Statistically significant values are given in bold.

Abbreviations: A, activity; FEF_25−75_, forced expiratory flow at 25−75%; FEV1, forced expiratory volume in 1 s; FFS, Fatigue Severity Scale; FVC, forced vital capacity; I, impact; MEP, maximal expiratory pressure; MIP, maximal inspiratory pressure; mMRC, modified Medical Research Council; PEF, peak expiratory flow; S, symptoms; SGRQ, St Georges Respiratory Questionnaire; T, total; % pred, % predicted; 6MWD, 6‐min walking distance.

* *p* < 0.05.

** *p* < 0.001.

**TABLE 3:** Comparison of pulmonary function and respiratory muscle strength before and after treatment

	Study group (*n* = 30)		*p*	Control group (*n* = 30)		*p*
	Pre mean ± SD	Post mean ± SD		Pre mean ± SD	Post mean ± SD	
FEV_1_, % pred	34.6 ± 7.33	40.1 ± 5.26	**0.040** *	36.4 ± 8.12	37.9 ± 10.2	0.152
FVC, % pred	50.4 ± 10.6	54.7 ± 12.0	**0.038** *	51.1 ± 11.6	52.9 ± 10.7	0.103
FEV_1_/FVC	56.2 ± 9.15	59.3 ± 8.63	**0.007** **	58.2 ± 12.4	59.8 ± 10.6	0.158
PEF, %	36.8 ± 14.7	39.4 ± 12.5	0.115	37.6 ± 15.2	38.8 ± 15.6	0.304
FEF_25−75_, %	19.8 ± 10.1	20.5 ± 9.85	0.256	20.1 ± 11.4	20.4 ± 11.8	0.865
MIP, cmH_2_O	55.5 ± 14.6	92.1 ± 12.7	**<0.001** **	56.8 ± 13.8	71.5 ± 14.6	**0.025** *
MIP, % pred	60.8 ± 18.7	102.1 ± 16.5	**<0.001** **	61.2 ± 16.7	80.3 ± 15.2	**0.016** *
MEP, cmH_2_O	85.1 ± 17.5	122.6 ± 18.9	**<0.001** **	88.1 ± 18.3	105.8 ± 19.3	**0.001** **
MEP, % pred	50.8 ± 20.1	66.7 ± 19.9	**<0.001** **	52.1 ± 21.5	59.8 ± 20.5	**0.003** **

*Note*: Statistically significant values are given in bold.

Abbreviations: FEF_25−75_, forced expiratory flow at 25−75%; FEV1, forced expiratory volume in 1 s; FVC, forced vital capacity; MEP, maximal expiratory pressure; MIP, maximal inspiratory pressure; PEF, peak expiratory flow; % pred, % predicted.

* *p* < 0.05.

** *p* < 0.001.

**TABLE 4:** Comparison of treatment effect on pulmonary function and respiratory muscle strength between the groups

	Study group mean change 95% CI	Control group mean change 95% CI	Response between the two groups mean difference 95% CI	Treatment affect *p*	Effect size Cohen's *d*
FEV_1_, % pred	4.18 (2.15 to 8.74)	0.64 (−3.25 to 6.29)	3.18 (2.62 to 3.73)	**0.014** *	0.783
FVC, % pred	4.06 (1.59 to 10.1)	0.72 (−3.98 to 7.60)	3.02 (2.48 to 3.55)	**0.023** *	0.865
FEV_1_/FVC	3.14 (1.43 to 7.75)	0.93 (−4.36 to 7.60)	2.05 (−0.06 to 4.16)	0.068	0.056
PEF, %	1.08 (−4.54 to 9.62)	0.78 (−6.82 to 9.14)	0.25 (−0.01 to 0.51)	0.628	0.060
FEF_25−75_, %	0.59 (−4.52 to 5.80)	0. 17 (−5.77 to 6.27)	0. 36 (−0.14 to 0.86)	0.732	0.132
MIP, cmH_2_O	35.5 (29.4 to 43.6)	13.8 (7.29 to 22.0)	20.12 (16.54 to 23.69)	**<0.001** **	1.235
MIP, % pred	40.2 (32.1 to 50.3)	18.96 (10.7 to 27.3)	20.05 (17.18 to 22.91)	**<0.001** **	1.156
MEP, cmH_2_O	36.3 (28.0 to 46.9)	17.4 (7.91 to 27.4)	17.8 (14.57 to 21.02)	**<0.001** **	0.965
MEP, % pred	14.7 (5.51 to 26.2)	7.15 (3.23 to 18.5)	6.94 (5.72 to 8.15)	**<0.001** **	0.895

*Note*: Statistically significant values are given in bold.

Abbreviations: FEF_25−75_, forced expiratory flow at 25−75%; FEV1, forced expiratory volume in 1 s; FVC, forced vital capacity; MEP, maximal expiratory pressure; MIP, maximal inspiratory pressure; PEF, peak expiratory flow; % pred, % predicted.

* *p* < 0.05.

** *p* < 0.001.

**TABLE 5:** Comparison of functional capacity, dyspnea, fatigue, and quality of life before and after treatment

	Study group (*n* = 30)		*p*	Control group (*n* = 30)		*p*
	Pre mean ± SD	Post mean ± SD		Pre mean ± SD	Post mean ± SD	
6MWT, m	395.1 ± 106.8	467.2 ± 112.4	**<0.001** **	386.7 ± 110.4	430.2 ± 114.5	**0.014** *
6MWT, % pred	59.4 ± 14.7	68.8 ± 15.2	**<0.001** **	58.7 ± 13.8	63.1 ± 14.1	**0.027** *
mMRC score	2.45 ± 0.72	1.69 ± 0.56	**<0.001** **	2.38 ± 0.62	1.97 ± 0.66	**<0.001** **
FFS	43.7 ± 12.7	30.1 ± 10.2	**<0.001** **	45.1 ± 11.8	35.3 ± 12.3	**<0.001** **
SGRQ‐S	62.1 ± 12.4	55.3 ± 11.7	**<0.001** **	62.3 ± 12.4	56.8 ± 10.8	**<0.001** **
SGRQ‐A	60.3 ± 14.8	45.6 ± 10.2	**<0.001** **	61.5 ± 15.1	51.3 ± 14.7	**<0.001** **
SGRQ‐I	40.5 ± 5.68	27.4 ± 6.79	**<0.001** **	40.7 ± 7.23	32.1 ± 6.78	**<0.001** **
SGRQ‐T	51.6 ± 7.74	40.8 ± 6.89	**<0.001** **	52.6 ± 6.77	45.2 ± 6.38	**<0.001** **

*Note*: Statistically significant values are given in bold.

Abbreviations: A, activity; FFS, Fatigue Severity Scale; I, impact; mMRC, modified Medical Research Council; S, symptoms; SGRQ, St Georges Respiratory Questionnaire; T, total; 6MWD, 6‐min walking distance; % pred, % predicted.

* *p* < 0.05.

** *p* < 0.001.

**TABLE 6:** Comparison of treatment effect on functional capacity, dyspnea, fatigue, and quality of life between the groups

	Study group mean change 95% CI	Control group mean change 95% CI	Response between the two groups mean difference 95% CI	Treatment affect *p*	Effect size Cohen's *d*
6MWT, m	66.1 (34.6 to 98.7)	42.5 (10.7 to −81.5)	20.8 (16.22 to 27.37)	**<0.001** **	0.915
6MWT, % pred	8.05 (4.38 to 11.1)	4.89 (1.82 to −8.64)	3.04 (2.5 to 3.57)	**<0.001** **	0.738
mMRC score	−0.78 (−1.10 to −0.43)	−0.32 (−0.74 to −0.07)	−0.37 (−0.45 to −0.13)	**<0.001** **	0.982
FFS	−13.4 (−19.1 to −7.23)	−9.7 (−15.5 to −3.02)	−2.95 (−3.65 to −2.14)	**<0.001** **	0.957
SGRQ‐S	−6.82 (−11.1 to −1.39)	−4.79 (−8.54 to −3.54)	−1.89 (−2.59 to −1.05)	**0.023** *	0.790
SGRQ‐A	−13.7 (−18.3 to −5.11)	−9.85 (−13.9 to −1.54)	−3.26 (−3.93 to −2.35)	**<0.001** **	0.896
SGRQ‐I	−12.5 (−15.3 to −8.85)	−7.14 (− 9.17 to −1.92)	−5.01 (−5.96 to −3.98)	**<0.001** **	0.875
SGRQ‐T	−10.2 (−14.5 to −7.00)	−7.38 (−10.7 to −4.00)	−2.17 (−2.75 to −1.56)	**<0.001** **	0.936

*Note*: Statistically significant values are given in bold.

Abbreviations: A, activity; FFS, Fatigue Severity Scale; I, impact; mMRC, modified Medical Research Council; S, symptoms; SGRQ, St Georges Respiratory Questionnaire; T, total; 6MWD, 6‐min walking distance; % pred, % predicted;

* *p* < 0.05.

** *p* < 0.001.

## 4 DISCUSSION

This study showed that the 12‐week IMT with MT program improves functional capacity, respiratory muscle strength, pulmonary function, and overall quality of life and reduces dyspnea and fatigue perception in patients with COPD.

The previous study shows that pulmonary rehabilitation does not provide clinically meaningful improvements in pulmonary function measurements. It has been determined that IMT can increase diaphragmatic velocity by increasing fibers II and reducing inspiratory time, and then can increase expiratory time, allowing reduction of hyperinflation.^25^ It has been shown in a meta‐analysis including 32 randomized controlled studies on the effects of IMT in COPD patients that IMT improves inspiratory muscle strength and endurance, functional exercise capacity, dyspnea, and quality of life.^7^ However, there are no studies in the literature evaluating the effectiveness of manual therapy with IMT.

Another study demonstrated improved FVC when pulmonary rehabilitation included MT.^26^ Under these conditions, changes in the elastic properties of the lungs and chest wall lead to an increase in static hyperinflation. In general, with increasing airway resistance and greater flow limitation, expiration towards relaxation volume becomes increasingly prolonged and the next inspiration begins before relaxation volume is reached. This places an extra load on the inspiratory muscles at end‐expiration: they have to overcome an additional “threshold” load related to the elastic recoil of the respiratory system before inspiratory flow commences. Furthermore, this occurs in the face of worsening mechanical advantage to the inspiratory muscles.^27^ Cruz‐Montecinos et al. reported that there was a significant reduction in TLC, ERV, and RV after a MT session and this study showed a positive effect of MT on hyperinflation.^11^ Another study demonstrated the immediate positive effects of MT on FEV_1_, FVC, respiratory muscle strength, and dyspnea perception.^24^ These results support the hypothesis that MT is effective in improving respiratory biomechanics and reducing respiratory work. One study evaluating the effect of exercise training together with manual therapy reported an increase in FVC and functional capacity^28^; however, another study, found that MT caused little change in pulmonary function values and did not affect pulmonary function values.^12^ In our study, after 12‐week IMT with MT, the average increment in MIP reached 35.5 cmH_2_O in the study group (effect size Cohen's *d*: 1.23, large effect size). According to the detailed meta‐analysis results reported by Gosslink et al., an increase of 13 cmH_2_O in MIP values reached minimal clinical significance level.^7^ So respiratory muscle strength was increased more in the presence of MT. Also, in our study, FEV_1_ and FVC values were improved with the addition of MT to IMT. Cohen's *d* values for FEV_1_ and FVC were 0.783 and 0.865, respectively, and according to these values, 12‐week IMT with MT had a large effect size on pulmonary function parameters. FEV1, FVC, and respiratory muscle strength were improved in the presence of MT. The application of MT techniques may increase chest wall and inspiratory muscle elasticity and reduce hyperinflation. Thus, the inspiratory muscle that was increased elasticity may have been performed with IMT.

A study investigating diaphragmatic mobility by M‐mode ultrasonography demonstrated diaphragmatic mobility loss in subjects with moderate to very severe COPD, with losses correlating with COPD severity improving after in‐patient pulmonary rehabilitation. The study found a positive correlation between diaphragm mobility and lung function in COPD.^29^ We think that application of the diaphragmatic release technique and IMT led to diaphragm mobility and improved pulmonary function.

In the present study, we assessed the benefit of IMT on dyspnea using the mMRC dyspnea scale. We found a decrease of dyspnea in the two groups, but a more remarkable decrease in symptoms in the study group (effect size Cohen's *d*: 0.92, large effect size). While the mean decrease in the mMRC score was 0.78, an approximate decrease of half this value was observed in the control group. Similarly, in a study investigating the effect of respiratory muscle training on respiratory muscle strength, dyspnea, functional capacity, and quality of life in patients with stage 2–4 COPD, the degree of dyspnea improved and the effect size Cohen's *d* was 1.67 for the baseline dyspnea index score.^30^ In another study comparing the effect of IMT and calisthenics‐breathing exercises that increase thoracoabdominal mobility in patients with COPD, it was shown that the mMRC score decreased and the effect size Cohen's *d* was 2.0.^31^ According to the researches the addition of IMT to pulmonary rehabilitation, there was no found significant improvement of dyspnea.^25^, ^32^ In our previous study, a single MT session reduced dyspnea perception.^24^ On the other hand in a comprehensive meta‐analysis reported, the decrease of dyspnea was almost clinically significant (−0.8 point) after IMT.^33^ The addition of MT to IMT increased inspiratory muscle strength and endurance more than IMT alone, and it could lower the oxygen cost of voluntary hyperpnea and relieve patients' degree of perceived dyspnea.^34^ Hence, our findings suggest including IMT and MT in COPD treatment will reduce dyspnea symptoms.

When the effect of IMT on the outcome of 6MWT was analyzed in the meta‐analysis, it was shown that IMT improved functional capacity with an average increase of 32 m.^7^ Similarly in our study, there was a significant improvement in functional capacity in both the IMT group and IMT‐with‐MT group, but the change in 6MWT distance was greater in the IMT‐with‐MT group. The average increment in the 6MWT distance was 66 m in the study group, and it has a large effect size (Cohen's *d*: 0.91). Chuang et al. reported a 48 m increase in the 6MWT distance in the experimental group, and the effect size of Cohen's *d* was 2.49 in their study for the 6MWT distance.^30^ In another study, after 4 months of IMT, the average improvement in the 6MWT distance reached 44 m in the IMT group and it has a moderate effect size (Cohen's *d*: 0.56).^31^ This difference may be due to improved chest biomechanics with MT, resulting in the reduction of chest wall rigidity and the achievement of the optimal length‐tension relationship for respiratory muscle. So, symptom severity and the work of breathing may decrease and functional capacity increase.

### 4.1 Limitations

The strengths of this study are that it is randomized and controlled and that the study and control groups had similar features and were homogeneous. The investigators who collected the data were blind to the study. Additionally, the study investigated the long‐term effect (12 weeks) of IMT and MT. It is the first to investigate the effectiveness of MT with IMT. The lack of static lung volume measurements to analyze the effect of MT and IMT on hyperinflation is a limitation of the study, as is the fact that GOLD stage 3 and 4 patients were included in the study to ensure homogeneity regarding the effects of the disease. However these effects can be investigated in different stages. Another significant limitation is that the patients in the IMT‐with‐MT group were seen much more frequently than the control group. That could have influenced performance‐dependent measurements such as MIP, MEP, 6MWD, and subjective measures such as FSS, SGRQ, and mMRC scores. In order to prevent this, no additional exercise was given in both groups during the study. In addition, the investigator who made the measurements did not know participant's group allocation. Patients' evaluations and treatments were performed by a different investigator. Treatments and measurements took place at different locations.

## 5 CONCLUSION

This randomized controlled single‐blind study demonstrated that the group of COPD patients receiving IMT with MT showed improved FEV_1_ and FVC values, improved functional capacity, increased respiratory muscle strength, improved pulmonary function, reduced dyspnea and fatigue perception, and improved quality of life compared with the group of patients treated with IMT alone. This study brings a new perspective to the application of IMT and its beneficial effects.

## CONFLICT OF INTEREST

The authors have no conflicts of interest to declare.

## AUTHOR CONTRIBUTIONS

All authors did the conception and design. YBÇ and GDYY planned the methods to generate the results, and YBÇ and GDYY provided oversight, responsible for organization and implementation, and writing of the manuscript. YBÇ, GDYY, and NDE were responsible for experiments, patient management, organization, or reporting data, responsible for statistical analysis, evaluation, and presentation of the results, YBÇ, GDYY, and NDE performed the literature search, and YBÇ, GDYY, and NDE were responsible for writing a substantive part of the manuscript. All authors did the final approval of the manuscript.

## ETHICS STATEMENT

Ethical approval was given by Fatih University Clinical Research Ethics Committee. The study was conducted according to the Declaration of Helsinki and its guidelines for Good Clinical Practice. Participants signed an informed consent form to participate in the study (Ethical approval number: B 30 2 FTH 0 20 00 00/1496).

## Data Availability

The data that support the findings of this study are available from the corresponding author upon reasonable request.
